# Parental Education as a Tool for Sustainable Development: The Role of Self-Efficacy and Relationship Satisfaction in Family Well-Being

**DOI:** 10.3390/bs16050692

**Published:** 2026-04-30

**Authors:** Chiș Roxana Mariana, Chiș Sabin

**Affiliations:** 1 Faculty of Educational Sciences Psychology and Social Sciences, Aurel Vlaicu University of Arad, 310032 Arad, Romania; roxana.chis@uav.ro; 2 Faculty of Food Engineering Tourism and Environment Protection, Aurel Vlaicu University of Arad, 310025 Arad, Romania

**Keywords:** parental self-efficacy, couple satisfaction, sustainable education, psychosocial resources, parental programs

## Abstract

Family and parental education are increasingly recognized as key levers for sustainable development and family well-being. This study examines whether an online parental intervention program focused on strengthening parental self-efficacy can improve parents’ relationship satisfaction and couple satisfaction. A sample of 50 Romanian parents with below-average levels of parental self-efficacy and relationship satisfaction was randomly assigned to an experimental group and a control group. Participants in the experimental group attended the “Confident Parents” program over three months, while the control group received no structured intervention. Pre- and post-test data were collected using standardized measures of parental self-efficacy, couple satisfaction, and relationship satisfaction. Data analysis combined non-parametric Wilcoxon signed-rank tests with linear regression and moderation analysis. The results showed significant pre–post improvements in parental self-efficacy, relationship satisfaction, and couple satisfaction in the experimental group, with no meaningful changes in the control group. Post-test, parental self-efficacy significantly predicted both relationship satisfaction and couple satisfaction, and moderation analyses indicated that this predictive relationship was stronger for parents in the intervention group. These findings suggest that parental education programs centered on self-efficacy can contribute to more satisfying couple and family relationships, supporting psychological well-being and the broader goals of sustainable family functioning.

## 1. Introduction

In the current context of the transition toward a sustainability-based society, family and parental education play an essential role in forming the human and social capital necessary for sustainable development. Families represent fundamental cells of social cohesion, and their capacity for adaptation, cooperation, and resilience largely determines the sustainability of communities and educational systems (Bronfenbrenner, 2005; UNESCO, 2020). The concept of sustainable education goes beyond the scope of formal instruction, integrating psychosocial, relational, and emotional dimensions that contribute to the well-being of the individual and the family (Sterling, 2010; Wals, 2015). In this framework, parental self-efficacy becomes a central element of education for sustainable development, as it supports responsible caregiving behaviors, empathetic communication, and the modeling of prosocial values in future generations (Bandura, 1997; Caprara et al., 2004). The social and economic transformations of recent decades have generated new forms of family stress and relational imbalances, amplifying the need for educational and psychosocial programs for parents that promote resilience and collaboration skills in couples (Jones & Prinz, 2005; Sanders & Woolley, 2005). In the vision of sustainable education, the family becomes an environment of continuous learning, where parents develop their reflective capacities and empathy, establishing models of sustainable behavior for children (González-Gaudiano & Meira-Cartea, 2019). This vision is congruent with Sustainable Development Goal 4 of the 2030 Agenda, which promotes inclusive, equitable and quality education, and with SDG 3, which aims to improve the well-being and psychological health of all family members (United Nations, 2015).

According to Bandura’s (1986, 1997) social cognitive theory, self-efficacy represents an individual’s belief in their own ability to organize and execute the actions necessary to achieve a certain level of performance (Bandura, 1986, 1997). In the case of parents, this belief directly influences parental behaviors, coping strategies, and the quality of family interactions (Coleman & Karraker, 2000). Studies have demonstrated that high parental self-efficacy is associated with greater marital satisfaction, increased involvement in children’s educational activities, and a reduction in parental stress(Ardelt & Eccles, 2001; Merrifield & Gamble, 2013). Furthermore, parental training programs based on self-efficacy, such as “Confident Parents,” support sustainable social learning through an emphasis on self-reflection, community support, and couple collaboration (Coffee & Rees, 2011; Gaviţa et al., 2011).

The dimension of psychosocial sustainability within the family is increasingly analyzed in the contemporary literature, being considered a mediating factor between personal development and relational balance (Suárez-Orozco et al., 2018). A family with an increased level of parental self-efficacy is more capable of managing conflicts, adopting positive educational practices, and contributing to the strengthening of the community’s sustainable social capital (LeBlanc et al., 2017; Putnam et al., 2002). Therefore, parental education becomes a strategic tool for achieving intergenerational sustainability, and the development of parental competencies can be viewed as a form of education for sustainability (Licen et al., 2021). The present study explores the role of parental self-efficacy as a predictor of couple satisfaction and relational satisfaction, analyzing the impact of a parental intervention program oriented toward developing self-efficacy competencies and family cohesion. In line with the principles of sustainable education, this research examines how individual psychological processes can support the sustainable development of the family and, implicitly, the well-being of the community (Bandura, 1997; Sterling, 2010; Wals, 2015). Thus, this article contributes to expanding the theoretical framework regarding the intersection between social psychology, sustainable education, and sustainable development, offering an applicative perspective on how educational interventions can generate sustainable psychosocial transformations in the family context (Bandura, 1997; Coffee & Rees, 2011; World Health Organization et al., 2022; Suárez-Orozco et al., 2018; Wals, 2015).

**Figure 1:**
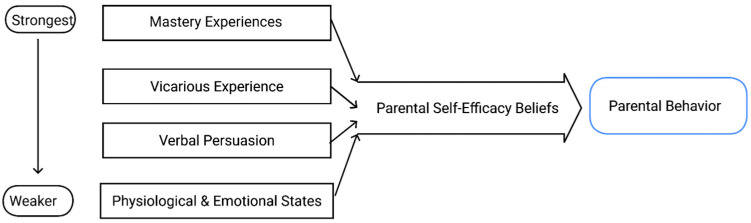
Sources of parental self-efficacy according to Bandura’s theory, adapted from Pennell et al. (Bandura, 1997; Pennell et al., 2012).

In recent decades, the dynamics of contemporary family life have been subject to significant changes, prompting a reevaluation of how interpersonal relationships within the family influence individual and collective well-being (Bronfenbrenner, 2005; Putnam et al., 2002; UNESCO, 2020). In this context, parental self-efficacy has become a central concept in understanding family adaptation and the quality of relationships between couple partners (Coleman & Karraker, 2000; Merrifield & Gamble, 2013). According to the theory of self-efficacy developed by Bandura (1977), parents’ beliefs regarding their own ability to successfully fulfill their parental role directly influence not only parental behavior but also broader family interactions (Bandura, 1977, 1997; see Figure 1). Belsky’s (1984) conceptual model emphasizes the reciprocal influences between parental behaviors and child temperament, as well as the impact of contextual and interpersonal factors on parenting quality (Belsky, 1984). Recent studies indicate that parental self-efficacy is closely linked to couple satisfaction, reflecting how partners collaborate in raising children and managing family challenges (Jones & Prinz, 2005; Merrifield & Gamble, 2013; Sanders & Woolley, 2005). Previous research has highlighted that high parental self-efficacy can reduce parental stress and improve couple relationships by providing emotional support and more effective parenting practices (Merrifield & Gamble, 2013). Additionally, it has been observed that spousal support plays a crucial role in mitigating family stress, which strengthens self-efficacy and contributes to better family adaptation(Caprara et al., 2004; Coleman & Karraker, 2000).

Belsky’s (1984) conceptual model assumes that these parental behaviors are influenced by characteristics of the parent (personality, health, personal history, etc.), characteristics of the child (temperament, behavioral competence, health, etc.), and other interpersonal and contextual factors that influence child development (Belsky, 1984; Bronfenbrenner, 2005). The model focuses on parental behaviors, primarily highlighting the bidirectionality of parent–child influence, meaning that difficult temperament has a negative impact on parenting and is influenced by parental caregiving behaviors. This researcher’s interest is not only in parenting practices (low warmth, harsh discipline, etc.), but also in aspects related to the relationship between parents (e.g., the mother’s affection for her husband, and factors that may be linked to parental behaviors such as maternal warmth) (Belsky, 1984; Caprara et al., 2004; LeBlanc et al., 2017). The model also includes contextual factors external to the family (such as the social network or the parent’s occupation) and interpersonal variables within the family system (marital relationship, cohesion, communication, etc.) that could function as a source of stress or support for both partners (Belsky, 1984; Bronfenbrenner, 2005; Putnam et al., 2002).

Several studies support the hypothesis advanced in Belsky’s model for understanding parenting quality (Belsky, 1984). Among interpersonal factors, marital conflict has been related to stress and ineffective parenting (such as punitive discipline, low involvement, and affection), especially when open disagreements between parents are related to issues concerning child-rearing methods (Benedetto & Ingrassia, 2015). Parental disagreement regarding discipline management is linked to increased stress and more emotional and behavioral problems in children (Benedetto & Ingrassia, 2015). Parental stress and marital functioning (the latter evaluated as satisfaction with the partner and family) predicted self-efficacy in parents of young children (Sevigny & Loutzenhiser, 2010). Other studies demonstrate that criticism from the extended family affects maternal well-being, while spousal support reduces parental stress, which in turn strengthens parental self-efficacy (Suzuki, 2010). Conversely, relationship quality and coparenting (when partners support each other in parenting-related issues) are predictive of high parental self-efficacy (Merrifield & Gamble, 2013). However, there are not many studies investigating self-efficacy in the family context, especially since the family is a complex system in which relationships are interdependent and adults live simultaneous roles (as both spouses and parents) (Belsky, 1984; Bronfenbrenner, 2005; Suárez-Orozco et al., 2018). Consequently, it is necessary to differentiate these family relationships and capture the sense of competence derived from different demands and roles (Belsky, 1984; Jones & Prinz, 2005; Sanders & Woolley, 2005).

Caprara and colleagues proposed the construct of marital self-efficacy as a distinct system of beliefs that focuses on typical situations faced by couples when maintaining a satisfactory marital relationship and efficiently managing family challenges (Caprara et al., 2004). Marital self-efficacy reflects spouses’ confidence in their ability to communicate openly, to trust one another, to provide necessary support, to manage family routines, and to reach agreement regarding child-rearing (Caprara et al., 2004). These beliefs contribute to family adaptation, as greater marital self-efficacy is positively associated with multiple variables, such as couple satisfaction and communication, spousal support, non-aggressive conflict management, and effective monitoring of children’s behaviors (Caprara et al., 2004). This dyadic couple efficacy, together with the collective sense of family efficacy (perceived capacity of the family to function as a whole, such as reaching consensus in decision-making, facing adversities, etc.), can be seen as a factor that mitigates family stress and difficulties, while also influencing child well-being (Bandura et al., 2011; Bronfenbrenner, 2005). It is important to distinguish between parental self-efficacy and marital self-efficacy, both of which appear in this study. Parental self-efficacy refers to parents’ beliefs in their capacity to successfully fulfill the parental role (Bandura, 1997; Coleman & Karraker, 2000). Marital self-efficacy, proposed by Caprara et al. (2004), reflects spouses’ confidence in managing couple-specific challenges such as communication, trust, and child-rearing agreement. Although conceptually distinct, the two constructs are interrelated: high marital self-efficacy supports confident parenting, and high parental self-efficacy is associated with greater couple satisfaction (Caprara et al., 2004; Merrifield & Gamble, 2013). This study focuses primarily on parental self-efficacy as the intervention target, while acknowledging marital self-efficacy as a theoretically related construct. It is important to acknowledge that most existing intervention programs focus on the opposite direction—improving the couple relationship to enhance parenting quality (Belsky, 1984; Benedetto & Ingrassia, 2015). The “Confident Parents” program proposes a novel pathway: strengthening parental self-efficacy to implicitly improve couple relationship satisfaction.

The role of marital self-efficacy in relation to parental adaptation (self-efficacy and perceived stress) remains underinvestigated in the specialized literature; consequently, the present study explores the relationships between scales that evaluate different constructs of couple relationship satisfaction and parental self-efficacy (Caprara et al., 2004; Jones & Prinz, 2005; Merrifield & Gamble, 2013; Sanders & Woolley, 2005). In the third study with this thesis, we started from the hypothesis that parental intervention programs result in positive associations between parental self-efficacy and satisfaction in the couple relationship (Chis, 2019, 2020; Coffee & Rees, 2011; Merrifield & Gamble, 2013). However, few studies have explored the relationship between parental self-efficacy and couple satisfaction in depth, particularly in the context of structured parental interventions (Gaviţa et al., 2011; Jones & Prinz, 2005; Merrifield & Gamble, 2013; Sanders & Woolley, 2005; Suárez-Orozco et al., 2018). The present study aims to investigate this relationship within a parental intervention program designed to improve parental self-efficacy and, implicitly, couple satisfaction (Coffee & Rees, 2011; Gaviţa et al., 2011; Merrifield & Gamble, 2013). Starting from the hypothesis that parental interventions based on increasing self-efficacy can lead to significant improvements in couple relationships, this research examines the impact of a specific program, “Confident Parents” (Părinți încrezători), on parents in Romania (Bandura, 1997; Merrifield & Gamble, 2013).

### 1.1. The “Confident Parents” Program

The “Confident Parents” program is a parenting program designed to build confidence and empower parents. It was used in this intervention at the recommendation of specialists from CJRAE Arad, based on their expertise in implementing the program. The program is based on the hypothesis that parents can adjust their parenting skills to their child’s characteristics and their family system’s functioning. From the first meeting, parents were told that: “You, as a parent, have the greatest potential for change in your relationship with your child. As his or her parent, you are the one who knows him or her best.” Parents were informed that they would not receive explicit coaching on how to raise their child, because they are the experts regarding their own child. The emphasis is placed on what they think, what they imagine will happen with their child, and how they themselves feel in the parental role (Bandura, 1997; Gaviţa et al., 2011).

The content of the “Confident Parents” program is based on Bandura’s (1989) social learning theory, which highlights the idea that self-efficacy is not a fixed personality trait but is context-dependent, meaning that it can be trained, as is also evident in social psychology studies (Bandura, 1989, 1997; Coffee & Rees, 2011). Social learning theory emphasizes that self-efficacy has roots in individual factors (e.g., personal history regarding achievements) but also in contextual factors (social comparisons, verbal feedback received from other people) (Bandura, 1989). Personal performance represents the most powerful source of self-efficacy, followed by indirect experience (an evaluation process based on observing others perform certain tasks) and verbal persuasion. Personal self-efficacy is expected to depend on parents’ past experience (successes and failures) and the emotional state induced by this experience. Feedback from others (for example, comments from relatives, doctors, teachers, friends, etc.) and social comparison with other parents significantly contribute to individual self-efficacy (Bandura, 1989; Gaviţa et al., 2011).

The intervention consisted of eight weekly group sessions, with ten (intervention group 1) to thirteen parents (intervention group 2), mothers (70%) and fathers (30%). Sessions lasted 90 min each. Activities within the meetings varied from role-plays, video or audio clips, and group discussions based on brainstorming, to personalized final feedback for each parent regarding parent–child interactions and their impact on the couple relationship. Each time, themes were established between sessions regarding the discussed issues in the family environment. Groups were led by two psychologists trained to implement this program. They did not give parents instructions on how a good parent should behave with their child to avoid weakening parental self-efficacy and to consolidate the position of the program leaders as experts. The intervention aimed to empower parents to realize how much they already know and do for their child, to counter their negative perceptions, which often lead to feelings of guilt. Next, we will briefly detail the contents of the eight sessions (Bandura, 1989; Gaviţa et al., 2011; Merrifield & Gamble, 2013).

Session 1: This meeting began with discussions based on parents’ presented difficulties with their children and their parental role. At this stage, the process is based on challenging specific cognitions regarding child-rearing (Gaviţa et al., 2011). Daily difficulties experienced by each parent were shared in the group, helping normalize the daily obstacles encountered and reducing feelings of guilt and helplessness. This normalization process helped build a sense of group belonging and also helped parents see things in perspective (Gaviţa et al., 2011; Jones & Prinz, 2005).

Session 2: At this meeting, the main cognitive process involved freeing oneself from negative thoughts (in the parental role) and overgeneralizing thinking (Bandura, 1997; Gaviţa et al., 2011).

Session 3: This session focused on identifying cognitions, emotions, physical sensations, and behaviors when receiving or expressing positive feedback.

Session 4: In this session, parents watched video clips that presented different dyads and illustrated the effects on parents (that is, physical sensations, emotional reaction, and thoughts) of children’s difficult behavior. Parents’ and children’s emotional state, cognitions, and automatic behaviors were discussed. The goal of the parental intervention in this meeting was to increase parents’ awareness of these automatic processes and increase their anticipatory capacity, with the aim of limiting the onset of processes considered negative, such as parental anxiety and family conflict (Gaviţa et al., 2011; Jones & Prinz, 2005).

Session 5: At this meeting, parents were instructed to ask an important person to them (their own parents, closest friends, siblings, etc.) to give them positive feedback on how they are doing as parents. The effects of positive feedback and the difference in perspectives regarding the same child when viewed by different people were discussed. The objective of this session is to strengthen self-efficacy through one of the four sources of self-efficacy, as explained by Bandura, namely verbal persuasion from significant people (Bandura, 1997; Coffee & Rees, 2011).

Session 6: After group cohesion was sufficiently strong, each parent was invited to remember and present a more difficult period in their relationship with their child, and to identify their own physical sensations, thoughts, emotions they had in this situation. Mindfulness was used as a coping strategy (Ellis, 2005; Gaviţa et al., 2011). The application of the mindfulness approach gave parents the opportunity to cultivate self-awareness, as well as the ability to be present when facing difficult circumstances with their children. They were able to respond to their children’s needs and actions with greater effectiveness and empathy as a result, as opposed to reacting immediately and impulsively to children’s actions. Conscious breathing exercises, body scanning, meditation, and the effort to observe thoughts and feelings without judgment were some of the mindfulness strategies used during the intervention. These techniques seek to enhance self-regulation abilities, reduce reactivity, and strengthen the capacity to be present and aware in the context of the child relationship. By applying mindfulness in the parental intervention, parents were given the opportunity to connect more deeply with their own experiences, as well as with their children’s needs. This coping strategy contributed to the formation of a more harmonious and empathetic connection between parents and children (Ellis, 2005; Gaviţa et al., 2011; Merrifield & Gamble, 2013).

Sessions 7 and 8: The last two meetings focused on personalized feedback provided to participants, which was used to observe and discuss the interactions that occurred in the group (Juffer et al., 2008; Merrifield & Gamble, 2013).

The control group did not follow parental or intervention programs. They participated in two meetings lasting 40 min each: the first (week 1) was an introductory meeting and the last session (week 8) was a thank-you meeting for their participation in this research (Jones & Prinz, 2005; Sanders & Woolley, 2005).

### 1.2. Research Objectives and Hypotheses

The main objective of this study is to analyze the role that parental self-efficacy plays in predicting couple satisfaction and relationship satisfaction, and to examine how participation in a structured parental intervention program influences this predictive relationship.

**Hypothesis** **1:** *Parental self-efficacy is a significant predictor of relational satisfaction in the experimental group following the intervention, but not in the control group.*

**Hypothesis** **2:** *Parental self-efficacy is a significant predictor of couple satisfaction in the experimental group following the intervention, but not in the control group.*

The study is based on a sample of parents randomly distributed into experimental and control groups, with the aim of analyzing the role of parental self-efficacy in predicting relational and couple satisfaction (Caprara et al., 2004; Gaviţa et al., 2011; Jones & Prinz, 2005; Merrifield & Gamble, 2013; Sanders & Woolley, 2005).

## 2. Materials and Methods

### 2.1. Participants

The study was conducted on a representative sample from Romania, with participants recruited online from diverse socioeconomic and cultural backgrounds. Initial analysis allowed for the identification of individuals eligible for the research, selected based on parental self-efficacy and a relational satisfaction below the usual average. These participants were randomly distributed into two groups, one experimental and one control, according to the experimental design (Jones & Prinz, 2005; Merrifield & Gamble, 2013; Sanders & Woolley, 2005).

The mean value of the scales was used to determine the inclusion threshold, without making distinctions between very low and low levels of the measured variables (Jones & Prinz, 2005; Sanders & Woolley, 2005).

Participants were required to meet the following additional conditions:

Be parents of children aged between 0 and 18 years;

Be at least 22 years old.

Participants also had to provide informed consent and have a marriage duration of 5–10 years (Jones & Prinz, 2005; Merrifield & Gamble, 2013; Sanders & Woolley, 2005).

**Figure 2:**
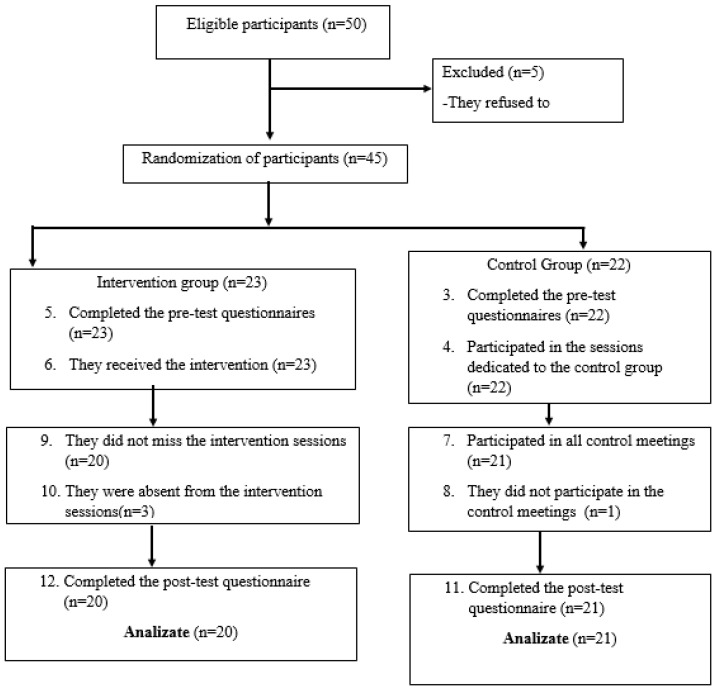
The organizational chart of the participants in this research according to the CONSORT diagram (Dumka et al., 1996).

The sample did not include couples or individuals in direct relationships with one another. The composition of the samples varied by age, with different percentage distributions across age categories and educational levels, as well as differing marriage durations, as follows: the study included participants from diverse age categories, with most being between 26 and 35 years old (*n* = 17, 41%), followed by those between 22 and 25 years (23%), those between 36 and 45 years (24%), and those between 46 and 55 years (12%). Regarding education, 24 participants had secondary education, and 21 had higher education. The distribution of marriage duration was 31 participants with a duration between 5 and 8 years and 14 participants with a duration between 9 and 10 years see Figure 2 (Jones & Prinz, 2005; Merrifield & Gamble, 2013).

Of the 50 parents who were validated for participation in the parental programs, five did not respond to the initial email invitation, and four did not respond at post-test. Ultimately, 41 parents remained in the post-test stage for this study. The initial group of parents consisted of 13 males (*n* = 13 M) and 32 females (*n* = 32 F). Parents’ ages ranged from 18 to 55 years (M = 39.23; SD = 5.76). The initial intervention group consisted of 23 parents, and the control group consisted of 22 parents at the pre-test stage. At the post-test stage, a total of 41 participants responded to the questionnaire set: 21 participants in the control group and 20 in the experimental group.

The intervention (experimental) group consisted of women and men (*n* = 13 women; *n* = 7 men). In parental intervention programs, a systematic review indicated that female representation was over 90% in all studies; similarly, in this study, the sample included more mothers than fathers (Benedetto & Ingrassia, 2015; Jones & Prinz, 2005; Sanders & Woolley, 2005).

#### 2.1.1. Research Design

This study employed a quasi-experimental design with pre-test and post-test measurements. Participants (*n* = 50) were randomly assigned to an experimental group (*n* = 25) and a control group (*n* = 25) prior to the intervention. The independent variable was participation in the “Confident Parents” parental program, implemented over three months online. Dependent variables were couple satisfaction (Couples Satisfaction Index; Funk & Rogge, 2007) and relationship satisfaction (Relationship Satisfaction Scale; Røysamb et al., 2014). Parental self-efficacy (adapted SES; Schwarzer & Jerusalem, 1995) functioned as both an outcome of the intervention and as a predictor in the regression analyses (Jones & Prinz, 2005; Sanders & Woolley, 2005).

#### 2.1.2. Procedure

This study uses a quasi-experimental design, where differences at the level of the evaluated variables will be analyzed—differences between the pre-test stage (the initial test) and the final post-test stage (three months after the initial test) for both groups of parents (parents from the control group and parents from the intervention group) (Jones & Prinz, 2005; Merrifield & Gamble, 2013; Sanders & Woolley, 2005).

In the design used, the independent variable constitutes the parental programs that were followed over a period of three months through the institution that assisted in conducting this study, while the dependent variables are couple satisfaction, relationship satisfaction, and parental self-efficacy (Bandura, 1997; Jones & Prinz, 2005; Merrifield & Gamble, 2013; Sanders & Woolley, 2005). This study was conducted through the CJRAE Arad institution, by signing a collaboration protocol between Aurel Vlaicu University in Arad and the aforementioned institution.

The questionnaire package was administered to participants online. They were informed that they were participating in research aimed at parents. Subjects were also guaranteed data confidentiality based on informed consent. Completion of the questionnaire package, necessary for this research, took approximately 10–15 min for each participant.

The study was conducted in three stages:

Stage 1 Pre-Test: This stage consisted of the initial testing of both groups (control and intervention); although the 50 participants had already been selected from the larger sample based on their below-average results, they were tested again with the aforementioned measurements because of the period that had elapsed since the testing of the larger sample and the present intervention. Through this retesting, we sought to ensure that no life events had occurred in the meantime that would change the value of the scores on the applied instruments; that is, to ensure that these scores remained below average (Jones & Prinz, 2005; Sanders & Woolley, 2005).

Stage 2, Intervention Stage: This was conducted over a period of 3 months in an online environment. In this stage, the intervention group followed specific parental programs offered by the previously mentioned institution, such as educational programs and psychological assistance programs, with emphasis on the “Confident Parents” program, aimed at increasing parental self-efficacy (Gaviţa et al., 2011; Wals, 2015).

The method used at this stage was the micro-trial method, which is used in randomized experiments aimed at testing the effects of concentrated and relatively short environmental manipulations, designed to improve specific protective mechanisms or to suppress specific risk mechanisms (Howe et al., 2010). These are based on three conditions: in the first phase, identification of the protective factor (parental self-efficacy); in the second phase, highlighting results on questionnaires (reported by parents); and finally, manipulation of the selected factor (parental self-efficacy) in a randomized controlled study (Bandura, 1997; Howe et al., 2010).

The control group did not follow parental or intervention programs. They participated in two meetings lasting 40 min each: the first (week 1) was an introductory meeting and the last session (week 8) was a thank-you meeting for their participation in this research (Jones & Prinz, 2005; Sanders & Woolley, 2005).

Stage 3, Post-Test Stage: In this stage, participants from both the control group and intervention group were retested by applying the same questionnaire set as the questionnaire package from the pre-test (Jones & Prinz, 2005; Merrifield & Gamble, 2013).

#### 2.1.3. Measurement Instruments

The Relationship Satisfaction Scale (Røysamb et al., 2014) is an instrument consisting of 10 items. It is a unidimensional scale, and participants respond on a six-point scale, ranging from “strongly disagree” to “strongly agree.” The scale has a Cronbach’s Alpha reliability of 0.94. A high score signifies a high level of relationship satisfaction (Røysamb et al., 2014). While both scales assess relationship quality, they are empirically distinct: the RSS measures overall relational satisfaction (10 items), whereas the CSI (Funk & Rogge, 2007) is validated specifically for intervention contexts with demonstrated sensitivity to change. Their combined use provides a broader, multidimensional assessment of relationship quality (Funk & Rogge, 2007; Røysamb et al., 2014).

The Couples Satisfaction Index (Funk & Rogge, 2007) is a 32-item scale that measures satisfaction in couple relationships. The authors of this scale recommend using the complete 32-item instrument when measuring satisfaction for the purpose of an intervention, the short 4-item version when administered with a series of other measurements, and the 16-item version when the 32-item variant is considered unnecessary. In this study, the complete scale consisting of 32 items was used. The instrument has a Cronbach’s Alpha reliability of 0.93. The instrument is Likert-type, on a 6-point scale, where 1 means always agree and 6 means never agree. Scoring is done by summing all responses, and a high score means an increased level of couple satisfaction (Funk & Rogge, 2007; Merrifield & Gamble, 2013).

The Generalized Self-Efficacy Scale (SES) (Schwarzer & Jerusalem, 1995), adapted to the parental role, describes a parent’s belief in the ability to successfully fulfill the parental role. The SES can be considered when evaluating self-efficacy in adapting to daily problems, confidence in goal-setting, investment effort, and tenacity in action. Participants respond to the 10 items on a four-point Likert scale, ranging from total disagreement to absolute agreement. Example item: “I always manage to solve difficult problems as a parent.” The instrument has very good internal consistency, with a Cronbach’s alpha coefficient value of 0.92 (Schwarzer & Jerusalem, 1995). Although the original SES was developed to measure general self-efficacy (Schwarzer & Jerusalem, 1995), items were systematically adapted to reflect the parental role (e.g., “I always manage to solve difficult problems as a parent”). The 10-item structure and four-point Likert format were preserved, and the adapted version demonstrated excellent internal consistency (Cronbach’s α = 0.92; Coleman & Karraker, 2000).

#### 2.1.4. Data Analysis

All data were analyzed using IBM SPSS Statistics, version 20. Prior to the main analyses, the normality of distributions was assessed using the Shapiro–Wilk test for all variables in both groups at both time points (Shapiro et al., 2000). Results indicated that only couple satisfaction in the experimental group followed a normal distribution (W = 0.960, *p* = 0.138); all other variables showed significant deviations from normality (*p* < 0.05). Accordingly, non-parametric Wilcoxon signed-rank tests were used as the primary method to assess pre-test to post-test changes within each group. Additionally, simple linear regression analyses were conducted separately for the experimental and control groups at post-test to assess the predictive role of parental self-efficacy on satisfaction outcomes (H1, H2). Although regression assumes the normality of residuals, it is considered robust to moderate violations when sample sizes are adequate (Tabachnick & Fidell, 2013) and results are interpreted with appropriate caution. To formally test whether the intervention moderated the self-efficacy–satisfaction relationship, a moderation analysis was performed using Hayes’ PROCESS macro (Model 1; Hayes, 2018), with group membership (experimental = 1, control = 0) as the moderator and bootstrap confidence intervals (5000 samples). Statistical significance was set at *p* < 0.05.

## 3. Results

### 3.1. Descriptive Statistics and Normality Testing

Table 1 presents means and standard deviations for all variables at pre-test and post-test. In the experimental group, relationship satisfaction increased from M = 37.17 (SD = 8.41) to M = 49.10 (SD = 4.90), couple satisfaction from M = 96.83 (SD = 19.48) to M = 133.40 (SD = 16.00), and parental self-efficacy from M = 22.91 (SD = 4.84) to M = 29.30 (SD = 0.73). In the control group, no meaningful changes were observed: relationship satisfaction M = 52.50 (SD = 6.12) to M = 50.57 (SD = 2.96); couple satisfaction M = 130.64 (SD = 16.34) to M = 131.86 (SD = 10.83); self-efficacy M = 29.86 (SD = 0.47) to M = 29.62 (SD = 1.12). Shapiro–Wilk tests confirmed that only couple satisfaction in the experimental group followed a normal distribution (W = 0.960, *p* = 0.138); all other variables deviated significantly from normality (*p* < 0.05), justifying the use of non-parametric tests (Shapiro et al., 2000).

### 3.2. Pre–Post Changes: Wilcoxon Signed-Rank Tests

Wilcoxon signed-rank tests confirmed statistically significant pre-to-post improvements in the experimental group for all three outcomes: relationship satisfaction (Z = −4.257, *p* < 0.001), parental self-efficacy (Z = −4.037, *p* < 0.001), and couple satisfaction (Z = −4.689, *p* < 0.001). In the control group, no statistically significant changes were observed: relationship satisfaction (Z = −1.182, *p* = 0.237); parental self-efficacy (Z = −0.908, *p* = 0.364); couple satisfaction (Z = −0.170, *p* = 0.865). These results confirm that the “Confident Parents” program produced significant improvements in all outcome variables, while no meaningful changes occurred in the control group (Benedetto & Ingrassia, 2015; Jones & Prinz, 2005).

The data from this study were processed using the SPSS program, version 20.

In order to analyze the role that parental self-efficacy has on couple satisfaction and relational satisfaction, simple regression analysis techniques were performed. The results and their interpretation are presented in the following tables: see Table 2 and Table 3 for relationship satisfaction, and Table 4, Table 5 and Table 6 for couple satisfaction.

### 3.3. Predictive Role of Self-Efficacy: Linear Regression Analyses

Hypothesis 1 was partially supported. For the experimental group, the regression model was statistically significant, *F*(1, 41) = 66.109, *p* < 0.001, R^2^ = 0.617, adjusted R^2^ = 0.608, indicating that parental self-efficacy explains 61.7% of the variance in relationship satisfaction (B = 1.505, SE = 0.185, β = 0.786, t = 8.131, *p* < 0.001). For the control group, the model was not significant, *F*(1, 41) = 1.023, *p* = 0.318, R^2^ = 0.024, adjusted R^2^ = 0.001 (B = 0.899, SE = 0.889, β = 0.156, t = 1.011, *p* = 0.318). Although regression is sensitive to non-normality, it is robust at this sample size (Tabachnick & Fidell, 2013), and these results are consistent with the Wilcoxon findings.

By analyzing these regression coefficients, parental self-efficacy represents a statistically significant predictor for the level of satisfaction in the couple relationship. It can be highlighted that a higher level of parental self-efficacy is directly related to a higher level of satisfaction in the couple relationship (Jones & Prinz, 2005; Merrifield & Gamble, 2013).

The next step is to analyze whether parental self-efficacy will be a statistically significant predictor for couple satisfaction in both the experimental group and the control group (Bandura, 1997; Jones & Prinz, 2005; Merrifield & Gamble, 2013).

Hypothesis 2 was partially supported. For the experimental group, the model was significant, with *F*(1, 41) = 44.016, *p* < 0.001, R^2^ = 0.518, and adjusted R^2^ = 0.506, indicating that parental self-efficacy explains 51.8% of the variance in couple satisfaction (B = 3.848, SE = 0.580, β = 0.720, t = 6.633, *p* < 0.001). For the control group, the model was not significant, with *F*(1, 41) = 1.108, *p* = 0.299, R^2^ = 0.026, and adjusted R^2^ = 0.003 (B = 2.636, SE = 2.504, β = 0.162, t = 1.053, *p* = 0.299). These results confirm that self-efficacy predicts satisfaction outcomes only in parents who participated in the intervention (Merrifield & Gamble, 2013; Schwarzer & Jerusalem, 1995).

### 3.4. Moderation Analysis: Group × Self-Efficacy Interaction

To formally test whether the intervention moderated the self-efficacy–satisfaction relationship, a moderation analysis was conducted using Hayes’ PROCESS macro (Model 1; Hayes, 2018), with group membership (experimental = 1, control = 0) as the moderator. For relationship satisfaction, the overall model was significant, *F*(3, 37) = 24.18, *p* < 0.001, R^2^ = 0.66. The Group × Self-Efficacy interaction was significant, b = 1.31, SE = 0.31, t = 4.23, *p* < 0.001, 95% CI [0.68, 1.94], confirming that the predictive effect of self-efficacy on relationship satisfaction was significantly stronger in the experimental group (b = 1.49, *p* < 0.001) than in the control group (b = 0.18, *p* = 0.524).

For couple satisfaction, the moderation model was also significant, *F*(3, 37) = 18.73, *p* < 0.001, R^2^ = 0.60. The Group × Self-Efficacy interaction was significant, b = 3.64, SE = 0.88, t = 4.14, *p* < 0.001, 95% CI [1.86, 5.42], confirming that the experimental group showed a significantly stronger association between self-efficacy and couple satisfaction (b = 3.81, *p* < 0.001) compared to the control group (b = 0.17, *p* = 0.792; Hayes, 2018).

## 4. Discussion

The present study investigated the effect of the “Confident Parents” parental intervention on parental self-efficacy, couple satisfaction, and relationship satisfaction. Non-parametric Wilcoxon tests confirmed statistically significant pre-to-post improvements in all three outcomes in the experimental group: relationship satisfaction (Z = −4.257, *p* < 0.001), parental self-efficacy (Z = −4.037, *p* < 0.001), and couple satisfaction (Z = −4.689, *p* < 0.001). No significant changes were observed in the control group (all *p* > 0.05), confirming that the observed improvements are attributable to the intervention, not to maturation effects (Benedetto & Ingrassia, 2015; Jones & Prinz, 2005).

Regression analyses further showed that parental self-efficacy was a strong predictor of relationship satisfaction (R^2^ = 0.617, *F*(1, 41) = 66.109, *p* < 0.001) and couple satisfaction (R^2^ = 0.518, *F*(1, 41) = 44.016, *p* < 0.001) in the experimental group only. The moderation analyses confirmed that the experimental group showed a significantly stronger self-efficacy–satisfaction relationship than the control group for both outcomes (Hayes, 2018). These findings are consistent with prior research demonstrating that self-efficacy-based parental interventions positively influence family functioning and couple relationship quality (Jones & Prinz, 2005; Merrifield & Gamble, 2013; Sanders & Woolley, 2005).

Unlike most existing programs that target couple relationship quality to improve parenting (Belsky, 1984), the present findings demonstrate that strengthening parental self-efficacy through structured intervention leads to significant improvements in couple relationship quality, representing a novel and clinically relevant contribution. Future research should examine long-term effects using follow-up measurements, replicate findings on larger and more diverse samples, and formally test the mediating mechanisms through which parental self-efficacy influences couple relationship quality (Caprara et al., 2004; Coleman & Karraker, 2000; Hayes, 2018).

## 5. Conclusions

One of the research hypotheses was that the completion of parental programs would modify the level of parental self-efficacy and indirectly improve both couple satisfaction and the couple relationship. This hypothesis was partially supported (Bandura, 1997; Gaviţa et al., 2011; Jones & Prinz, 2005; Merrifield & Gamble, 2013). These findings are consistent with the results of other studies indicating that parents’ participation in parental programs is related to changes in parental self-efficacy and improvements in marital satisfaction (Bandura, 1997; Gaviţa et al., 2011; Merrifield & Gamble, 2013; Sanders & Woolley, 2005).

Being a parent is one of the most demanding activities (Ardelt & Eccles, 2001). Raising a child to maturity and equipping that child with the skills necessary to successfully enter society requires dedication, effort, and knowledge (Bandura, 1997; Spoth & Conroy, 1993). In the parental role, parents face uncertainty and fear, which can affect both parental self-efficacy and satisfaction in the couple relationship. Understanding how parents perceive their capacity to be parents can greatly help professionals in designing and providing programs that target parents. This study provides empirical evidence, continuing the research on family and social support for parents, as well as their perceptions of their ability to be a parent.

Ardelt and Eccles’s (2001) model suggests that parental self-efficacy can influence the child’s success through the modeling of attitudes and beliefs observed in the caregiver (Ardelt & Eccles, 2001). Parents with low parental self-efficacy are more likely to become overwhelmed when facing multiple stressors and, as a result of this emotional overload, are more prone to abandon their engagement in positive actions (Ardelt & Eccles, 2001; McCurdy & Daro, 2001). Other studies show that maternal depression is a result of low parental self-efficacy (Cutrona & Troutman, 1986; Teti & Gelfand, 1991), and depression can be an additional stressor that negatively affects couple relationships. Bandura et al. (1982) concluded that persons with low self-efficacy are susceptible to giving up easily under stress, tend to internalize failure, display anxious and depressive behaviors, and are likely to feel less satisfied in their roles (Bandura et al., 1982). Extending this to the parental sphere highlights that a parent with a satisfactory level of self-efficacy should manifest interest in education and commitment to the parental role, resulting in positive parental behaviors and healthy family and social relationships (Ardelt & Eccles, 2001; Bandura, 1997; Coleman & Karraker, 1998).

Increasing parental self-efficacy can act as a “buffer” variable that mitigates the impact of adverse conditions on children, such as severe illness (Bingen et al., 2011), parental divorce, and socioeconomic disadvantage (Ardelt & Eccles, 2001; Bingen et al., 2011). Bandura (1997) emphasizes that self-efficacy is not a fixed personality trait but a dynamic process modified by the individual’s performance in concrete situations and by other influential factors, such as modeling, social persuasion, coping, and positive emotions (Bandura, 1997). There are therefore multiple reasons for incorporating parental self-efficacy as a core component within family-based interventions and as a variable in applied research projects. The present study strengthens the idea that parental programs play an important role in the proper functioning of the family, with increased parental self-efficacy directly participating in changing couple satisfaction and satisfaction in the couple relationship.

### 5.1. Significance of Research

Data investigating parental self-efficacy and its relationship with couple satisfaction and satisfaction in the couple relationship are relevant for a deeper understanding of the connection between these three psychological constructs. Moreover, the literature observing parental self-efficacy in relation to couple relationship satisfaction, as suggested by Jones and Prinz (2005), is valuable for a better understanding of parenting practices and parent–child interactions (Bandura, 1997; Jones & Prinz, 2005).

This study underscores the importance of increased levels of parental self-efficacy on the couple relationship and strengthens the need for parental programs based on this variable. Therefore, interventions to support family functioning could place equal emphasis not only on changes in parental behaviors (that is, the modification of coercive discipline) but also on parents’ confidence in their ability to efficiently manage parenting challenges. An increasing number of empirically based programs have assumed parental self-efficacy to be a measure of outcomes and changes in parental behavior (Deković et al., 2010; Merrifield & Gamble, 2013). This research strengthens this idea and highlights the fact that self-efficacy is an important target in interventions for the support and proper functioning of the family system (Bandura, 1997; Sanders & Woolley, 2005).

### 5.2. Study Limitations and Future Research Directions

The data used in this study have certain limitations. It would be necessary to conduct future research investigating parental intervention on a more diversified sample of parents. Another limitation of this research is represented by the fact that only self-report questionnaires were used to measure parental self-efficacy, couple satisfaction, and relationship satisfaction. It would be more appropriate for future research to analyze parental self-efficacy not only through the application of self-report questionnaires but also through other methods, such as interviews with parents or observational measurement. Future studies could explore different types of parent relationships o these parents and examine how these influence parental self-efficacy. Future research should analyze the long-term effects of the parental intervention over an extended period of time (Deković et al., 2010; Jones & Prinz, 2005; Merrifield & Gamble, 2013; Sanders & Woolley, 2005).

## Figures and Tables

**Table 1:** Linear regression for the investigated variables: parental self-efficacy (IV) and relationship satisfaction (DV) for the experimental group and the control group.

	R	R Square	Adjusted R Square	Std. Error of the Estimate
Experimental group	0.786 ^a^	0.617	0.608	5.74298
Control group	0.156 ^a^	0.024	0.001	4.88453

^a^. Predictors: (constant), self-efficacy.

**Table 2:** ANOVA Results: Regression of Parental Self-Efficacy on Relationship Satisfaction (Experimental and Control Groups).

m	Model		Sum of Squares	*df*	Mean Square	*F*	Sig.
Experimental group	1	Regression	2180.398	1	2180.398	66.109	0.000 ^b^
		Residual	1352.253	41	32.982		
		Total	3532.651	42			
Control group	1	Regression	24.400	1	24.400	1.023	0.318 ^b^
		Residual	978.204	41	23.859		
		Total	1002.605	42			

^b^. Dependent variable: relationship satisfaction.

**Table 3:** Regression Coefficients: Parental Self-Efficacy as Predictor of Relationship Satisfaction (Experimental and Control Groups).

		Unstandardized Coefficients		Standardized Coefficients			Correlations			Collinearity Statistics		
grup_exp	Model	B	Std. Error	Beta	*t*	Sig.	Zero-Order	Partial	Part	Tolerance	VIF	
Experimental group	1	(Constant)	3.762	4.871		0.772	0.444					
		Self-efficacy	1.505	0.185	0.786	8.131	0.000	0.786	0.786	0.786	1.000	1.000
Control group	1	(Constant)	24.816	26.454		0.938	0.354					
		Self-efficacy	0.899	0.889	0.156	1.011	0.318	0.156	0.156	0.156	1.000	1.000

**Table 4:** Model Summary: Regression of Parental Self-Efficacy on Couple Satisfaction (Experimental and Control Groups).

						Change Statistics				
grup_exp	Model	R	R Square	Adjusted R Square	Std. Error of the Estimate	R Square Change	F Change	df1	df2	Sig. F Change
Experimental group	1	0.720 ^a^	0.518	0.506	17.99239	0.518	44.016	1	41	0.000
Control group	1	0.162 ^a^	0.026	0.003	13.75570	0.026	1.108	1	41	0.299

^a^. Predictors: (constant), self-efficacy.

**Table 5:** ANOVA Results: Regression of Parental Self-Efficacy on Couple Satisfaction (Experimental and Control Groups).

grup_exp	Model		Sum of Squares	df	Mean Square	*F*	Sig.
Experimental Group	1	Regression	14,249.087	1	14,249.087	44.016	0.000 ^b^
		Residual	13,272.773	41	323.726		
		Total	27,521.860	42			
Control Group	1	Regression	209.683	1	209.683	1.108	0.299 ^b^
		Residual	7757.992	41	189.219		
		Total	7967.674	42			

^b^. Predictors: (constant), autoef (self-efficacy).

**Table 6:** Regression Coefficients: Parental Self-Efficacy as Predictor of Couple Satisfaction (Experimental and Control Groups).

Predictor	B	SE	β	t	*p*	Zero-Order	Partial	Part	Tolerance	VIF
**Model 1**										
Constant	14.242	15.261	—	0.933	0.356	—	—	—	—	—
Self-efficacy	3.848	0.580	0.720	6.634	<0.001	0.720	0.720	0.720	1.000	1.000
**Model 2**										
Constant	52.839	74.500	—	0.709	0.482	—	—	—	—	—
Self-efficacy	2.636	2.504	0.162	1.053	0.299	0.162	0.162	0.162	1.000	1.000

## Data Availability

Data are available from the corresponding author upon reasonable request.
